# Exercise Intervention for Alzheimer’s Disease: Unraveling Neurobiological Mechanisms and Assessing Effects

**DOI:** 10.3390/life13122285

**Published:** 2023-11-30

**Authors:** Jianchang Ren, Haili Xiao

**Affiliations:** 1 Institute of Sport and Health, Guangdong Provincial Kay Laboratory of Development and Education for Special Needs Child, Lingnan Normal University, Zhanjiang 524037, China; 2 Institute of Sport and Health, South China Normal University, Guangzhou 510631, China; 3 Institute of Sport and Health, Lingnan Normal University, Zhanjiang 524037, China; xiaohl@lingnan.edu.cn

**Keywords:** exercise, physical activity, Alzheimer’s disease, molecular pathways, brain-derived neurotrophic factor, artificial intelligence, neuroimaging technologies

## Abstract

Alzheimer’s disease (AD) is a progressive neurodegenerative disease and a major cause of age-related dementia, characterized by cognitive dysfunction and memory impairment. The underlying causes include the accumulation of beta-amyloid protein (Aβ) in the brain, abnormal phosphorylation, and aggregation of tau protein within nerve cells, as well as neuronal damage and death. Currently, there is no cure for AD with drug therapy. Non-pharmacological interventions such as exercise have been widely used to treat AD, but the specific molecular and biological mechanisms are not well understood. In this narrative review, we integrate the biology of AD and summarize the knowledge of the molecular, neural, and physiological mechanisms underlying exercise-induced improvements in AD progression. We discuss various exercise interventions used in AD and show that exercise directly or indirectly affects the brain by regulating crosstalk mechanisms between peripheral organs and the brain, including “bone–brain crosstalk”, “muscle–brain crosstalk”, and “gut–brain crosstalk”. We also summarize the potential role of artificial intelligence and neuroimaging technologies in exercise interventions for AD. We emphasize that moderate-intensity, regular, long-term exercise may improve the progression of Alzheimer’s disease through various molecular and biological pathways, with multimodal exercise providing greater benefits. Through in-depth exploration of the molecular and biological mechanisms and effects of exercise interventions in improving AD progression, this review aims to contribute to the existing knowledge base and provide insights into new therapeutic strategies for managing AD.

## 1. Introduction

Alzheimer’s disease (AD) is one of the major challenges in the field of public health, characterized by a gradual decline in cognitive function and memory [1,2]. The pathological hallmarks of AD include the deposition of amyloid-beta (Aβ) proteins and the formation of neurofibrillary tangles due to abnormally phosphorylated tau protein, leading to neuronal damage and death [2,3,4]. There are approximately 44 million AD patients globally, and this number is expected to rise to 130 million by 2050 [5,6,7]. The pathogenesis of AD remains unclear, with current hypotheses including Aβ deposition, tau protein phosphorylation, cholinergic hypothesis, and inflammatory hypothesis, among others. Recent studies indicate that soluble Aβ oligomers are chiefly responsible for neurotoxicity [8]. In the brains of AD patients, abnormal aggregation of tau protein correlates with the severity of symptoms [9]. AD also involves significant loss of cholinergic neurons, leading to cognitive and behavioral disorders [10,11,12]. Inflammatory responses also play a role in AD, where Aβ may activate astrocytes to release neurotoxic inflammatory factors [13]. Apolipoprotein E4 (ApoE4) plays a crucial role in AD and other forms of neurodegenerative diseases, not only as a risk factor but also as a pathogenic factor. The APOE ε4 (*APOE4*) genotype may contribute to the maintenance of cognitive function in the absence of tau protein pathology, but once tau protein pathology emerges, ApoE4 promotes the formation of pathological structures and increases the risk of AD. The structural characteristics of ApoE4 also reveal potential therapeutic strategies, such as the use of drugs or other interventions to correct the structure of ApoE4, inhibit its protein cleavage, or protect mitochondrial function to alleviate or eliminate its negative effects. These treatment strategies need to consider the individual’s *APOE* genotype and the specific pathological state of the disease [14,15]. Currently, biomarkers for AD primarily include MRI, CSF analysis, and PET imaging [16,17]. Each of these detection methods has its strengths and limitations, and the advancement of artificial intelligence and neuroimaging techniques offers significant potential for the diagnosis and treatment of AD.

In terms of treatment, current clinical drugs used to treat AD, such as acetylcholinesterase inhibitors and N-methyl-D-aspartate receptor antagonists, can only delay the progression of AD to a certain extent and do not achieve a curative effect. Currently, there are still many drugs targeting synapses and neurotransmitters that are under research and development [18,19]. Non-pharmacological interventions are crucial for preventing the occurrence of AD. Standard care for AD encompasses both pharmacological and non-pharmacological interventions. Pharmacologically, cholinesterase inhibitors are the widely accepted first-line medications that are used to alleviate early symptoms and decelerate the progression of AD. For more advanced symptoms, NMDA receptor antagonists have also shown some efficacy in providing relief.

On the non-pharmacological front, cognitive stimulation activities, occupational therapy, and physical therapy have all demonstrated benefits in improving cognitive function and the ability to perform daily activities. Among these, regular exercise is considered a crucial component of non-pharmacological interventions. Exercise not only has the potential to enhance cognitive functions but can also reduce disease risk factors through the management of blood pressure, body weight, and other means. The low cost and ease of maintaining regular physical activity make exercise interventions particularly sustainable. Exercise promotes social interaction and can alleviate the psychological burden associated with caregiving. A variety of exercise types and intensities can yield positive effects, allowing for personalized intervention plans. Overall, exercise interventions can be effectively integrated with existing pharmacological and non-pharmacological treatments, creating a comprehensive and sustainable care plan for AD.

In recent years, a large number of studies have shown that scientific and regular exercise can effectively improve cognitive function and learning and memory abilities of the brain [20,21,22]. As a non-pharmacological intervention for preventing and treating AD, the biological mechanisms underlying the improvement of cognitive function in AD patients via exercise have been extensively demonstrated in clinical and animal experiments. Exercise can promote cerebral blood circulation and re-distribution of cerebral blood flow and enhance antioxidant effects by increasing the activity of enzymes and pro-inflammatory cytokines; exercise can promote neural regeneration and synapse formation by increasing the expression of vascular endothelial growth factor, brain-derived neurotrophic factor, and neurotrophic factors and reducing the aggregation of Aβ plaques, thereby improving the brain structures and neural circuits involved in cognition; exercise can also delay the pathological progression of AD by inhibiting the accumulation of hyper-phosphorylated tau protein or reducing the deposition of Aβ in the brain, but the specific mechanism is still unclear. The integrated biology of exercise suggests that exercise is a challenge to the homeostasis of the human body, breaking the original balance and triggering responses of cells, tissues, and organs. This multi-level integration creates a new dynamic balance for the organism. The close connection between the skeleton and the brain reveals the intrinsic correlation between the skeleton and AD. As an endocrine organ, the skeleton gradually secretes bone-derived factors such as glycoprotein sclerostin (SOST), osteocalcin (OCN), osteopontin (OPN), small glial cells, and hematopoietic stem cells, which regulate the physiological characteristics of AD in the brain through the blood–brain barrier and improve the metabolic process of AD. Exercise stimulates the endocrine function of the skeleton, regulates the secretion and expression levels of bone-derived factors, and finally slows down the pathological changes of AD and improves the cognitive function of AD through “bone–brain crosstalk”. As the main organ of the exercise system, skeletal muscle secretes active factors called “myokines”. Exercise can up-regulate the expression of brain-derived neurotrophic factors, irisin, and tissue proteinase B, and down-regulate the expression of muscle growth inhibitors, interleukin-6, and other factors. It can effectively inhibit the deposition of β-amyloid protein and act on the brain, affecting the structure and function of the central nervous system. Through “muscle–brain crosstalk”, it can prevent and alleviate AD. The gut microbiota–gut–brain axis immune pathway plays an important role in the occurrence and development of AD. Dysregulation of the gut microbiota and its products increases the production and deposition of Aβ in the brain through the gut–brain axis cross-seeding, damages the structure and permeability of the gut barrier, induces brain and peripheral inflammation reactions, and exacerbates AD pathology. Exercise, as a multi-target, low-injury, and easy-to-implement intervention, can directly or indirectly regulate the gut microbiota-gut–brain axis immune pathway by improving the structure and metabolites of the gut microbiota, thereby playing an important role in improving Aβ and inflammatory pathology mediated by gut microbiota.

Numerous studies have demonstrated that exercise improves symptoms of AD through various mechanisms. These mechanisms can interact with each other to promote healthy brain function and enhance mood [23,24,25,26]. The aim of this study is to analyze the neurobiological mechanisms underlying the relationship between exercise and AD, elucidate the potential mechanisms through which exercise improves AD, and evaluate the effectiveness of exercise interventions in treating AD. This will provide a new theoretical foundation and research perspective for the study of exercise and AD. Additionally, it will help develop more targeted exercise intervention programs for the effective management of AD.

## 2. Exercise Regulates Bone-Derived Factors to Improve Symptoms of Alzheimer’s Disease through “Bone–Brain Crosstalk”

The skeletal system serves both locomotive and important endocrine functions. Bone cells, osteoblasts, and bone marrow secrete bone-derived factors such as osteocalcin (OCN), sclerostin (SOST), and osteopontin (OPN) into the bloodstream. These factors are able to cross the blood–brain barrier and enter the brain. This establishes a communication pathway between bone and brain tissues, known as the “bone–brain crosstalk” mechanism. Through this mechanism, bone-derived factors in the circulation can regulate brain development and physiological processes. Specifically, they are involved in maintaining neuronal structure and function, enhancing synaptic plasticity, reducing neuroinflammation, and promoting cerebral blood vessel formation. In the context of Alzheimer’s disease, the bone–brain crosstalk aims to improve symptoms by inhibiting amyloid-beta (Aβ) plaque formation, promoting Aβ clearance from the brain, and enhancing cognitive function. Overall, it works to slow memory loss, cognitive decline, and progression of the disease.

**Figure 1:**
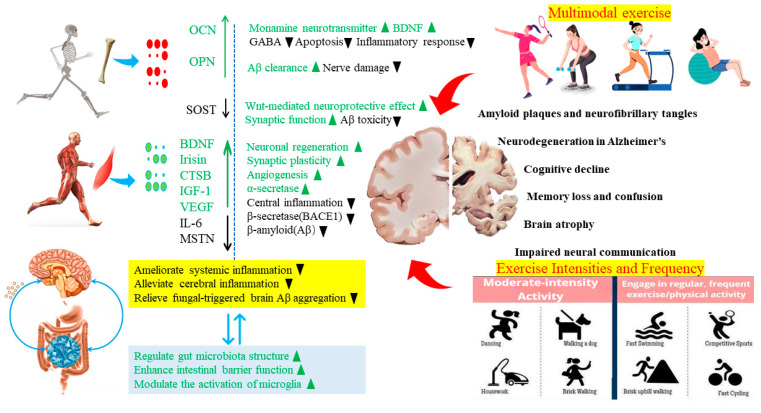
Mechanism of exercise-induced “periphery–brain crosstalk” in improving Alzheimer’s disease. Exercise stimulates the secretion of bone-derived factors, such as SOST, OCN, and OPN, by osteocytes, osteoblasts, and bone marrow. These factors enter the brain through the blood–brain barrier system, thereby regulating brain development and physiological function, improving AD symptoms, slowing down AD memory loss and cognitive decline, suppressing Aβ formation, promoting Aβ plaque clearance, and improving AD cognitive impairment. Exercise regulates the expression levels of neurotrophic factors, such as brain-derived neurotrophic factor (BDNF), irisin, interleukin-6 (IL-6), cathepsin B (CTSB), myostatin (MSTN), insulin-like growth factor-1 (IGF-1), and vascular endothelial growth factor (VEGF), through the stimulation of skeletal muscle. After entering the bloodstream, these muscle-related factors can cross the blood–brain barrier (BBB) and participate in maintaining neuronal structure and function, enhancing synaptic plasticity, reducing central inflammation, increasing brain vascularization, effectively inhibiting β-amyloid deposition, and improving the structure and function of the central nervous system. Exercise improves the structure of the gut microbiota and its metabolites in AD, directly or indirectly regulating the gut microbiota–gut–brain axis immune pathway. This leads to improvements in microbiota-mediated brain Aβ accumulation, enhancement of gut barrier function, reduction of intestinal permeability, and indirect improvement of peripheral inflammatory responses. Exercise also directly regulates peripheral immunity to suppress peripheral inflammatory responses, thereby targeting multiple points to improve peripheral inflammatory response-induced brain inflammation. It regulates the activation quantity and type of microglia and gut microbiota metabolites, directly or indirectly improving brain inflammation. Exercise plays an important role in improving the Aβ and inflammatory pathology mediated by the gut microbiota. **↑**, ▲: stimulatory effects; **↓**, ▼: inhibitory effects; **↓↑**: bidirectional regulation. The elements in the picture are all from the public Internet. https://www.shejihz.com/archives/67612/ (accessed on 24 November 2023). https://699pic.com/sucai/ (accessed on 24 November 2023).

1. OCN produced by osteoblasts enhances the synthesis and secretion levels of monoamine neurotransmitters (MN) while inhibiting the secretion and synthesis of γ-aminobutyric acid (GABA) [27], thereby improving learning, memory, and brain metabolic function. OCN also increases the secretion of brain-derived neurotrophic factor (BDNF), which subsequently improves the impact on neurodevelopment and function, reduces inflammatory responses, inhibits cell apoptosis, and suppresses anxiety and depressive behaviors. Exercise-induced skeletal stimulation can regulate OCN secretion levels, allowing it to accumulate in the brainstem, thalamus, and hypothalamus through the blood–brain barrier [28]. It binds specifically to neurons in the brain, influencing neurotransmitter synthesis and signal transmission. OCN further promotes the organism’s learning and memory abilities, increases neurotransmitter synthesis, and improves hippocampal development, thus enhancing cognitive function and inhibiting the development of anxiety emotions [29]. Additionally, OCN directly prevents neuronal apoptosis in the hippocampus, thereby protecting cognitive functions such as spatial learning and memory [30]. The binding of OCN and Gpr158 in CA3 pyramidal neurons of the hippocampus enhances the synthesis and secretion of neurotransmitters such as 5-hydroxytryptamine (5-HT), dopamine (DA), and norepinephrine (NE), while inhibiting GABA synthesis, thereby improving spatial learning and memory abilities [31]. The total osteocalcin (tOCN) content in the body primarily consists of carboxylated osteocalcin (cOCN) and undercarboxylated osteocalcin (uOCN), which is biologically active. Exercise stimulation can increase the overall activity level of uOCN. Additionally, exercise promotes the production of active uOCN by increasing muscle secretion of interleukin-6 (IL-6) [32,33]. Higher circulating OCN levels can significantly regulate the prevention of age-related cognitive decline. Exercise improves skeletal secretion capacity, increases circulating OCN levels in the body, and induces neuronal plasticity. Improved cognitive function can be achieved through OCN signaling [34,35,36,37].
2. The SOST, synthesized by osteocytes, binds to LDH receptor-related proteins 4/5/6 (Lrp4/5/6) to antagonize the Wnt/β-catenin signaling pathway. Intracerebral Wnt/β-catenin signaling is involved in maintaining neurogenesis, synaptic plasticity, and blood–brain barrier integrity. Wnt/β-catenin signaling regulates synaptic plasticity, and memory processes, inhibits neurotoxicity caused by Aβ, and participates in tau protein phosphorylation and learning and memory. Dysfunction of Wnt/β-catenin can lead to the production and aggregation of Aβ, thereby triggering the onset of AD [38,39]. Wnt/β-catenin signaling is considered a potential mechanism for treating AD. Among the mechanisms that affect brain Wnt/β-catenin signaling, Dickkopf-related protein 1 (Dkk1) can inhibit Wnt signaling by inducing LRP5/6, thereby blocking the Wnt signal-induced synaptic disassembly process [40]. Dkk1 is overexpressed in AD patients and in the brains of AD mice, and its expression levels can be effectively reduced through exercise [41]. Exercise stimulation activates the Wnt signaling pathway in APP/PS1 rats, improving synaptic dysfunction and promoting synaptic plasticity and neurogenesis in the hippocampus [42,43]. In the brain mechanism, Aβ activates glycogen synthase kinase-3β (GSK-3β) and reduces β-catenin activity, blocking the Wnt/β-catenin signaling pathway. Exercise attenuates the secretion of SOST from the bone, thereby effectively improving the occurrence and development of AD through the Wnt/β-catenin pathway. Research has found that the expression levels of SOST significantly decrease in 8-week-old mice after 5 weeks of exercise. In human studies, both highly active males and females exhibit lower levels of SOST secretion and circulation [44]. Exercise activates Wnt/β-catenin signaling in the brain, leading to an increase in β-catenin levels within brain cells, which tends to stabilize. Rats subjected to treadmill exercise for 30 min/day, 5 days/week, for a total of 12 weeks, show increased expression levels of Wnt3, reduced expression of GSK-3β, activation of the Wnt signaling pathway, increased neurogenesis, and alleviated memory loss associated with AD [45]. After exercise, the secretion of SOST from the bone decreases, resulting in a reduced amount circulating through the blood–brain barrier. This reduction weakens its binding with Lrp4/5/6, further activating the Wnt/β-catenin signaling pathway, ultimately promoting synaptic plasticity and neurogenesis, and mitigating AD levels and biological mechanisms [46].
3. OPN is a matrix cell immune regulator highly expressed by monocytes in the bone marrow, and it can regulate immune cell migration while responding to brain injuries [47]. Compared to chronic patients, AD patients have higher levels of OPN protein in their cerebrospinal fluid and plasma, suggesting that OPN plays a role in protecting neurons regulating brain diseases, and repairing neurodegenerative diseases [41]. OPN is involved in the process of brain remodeling, promoting myelination formation and regeneration [48]. Moderate-intensity (85% VO2 max) treadmill and weight-bearing running interventions for 5 weeks in 2-month-old male C57BL/6 mice can improve mouse bone mineral density (BMD), cortical bone mass, and osteogenic ability, as well as increase the expression and secretion levels of OCN and OPN in osteoblasts [49,50]. OPN enters the brain through the blood–brain barrier for regulation. OPN is mainly involved in AD neuron loss, degeneration, and the death of neurons. It plays a role in AD neuron abnormalities and re-entry into the cell cycle and/or myelin regenerative processes [51]. Studies have found that the increased expression of OPN is closely related to Aβ deposition in the cone neurons of AD patients and the brains of APP/PS1 mice [52]. In the process of OPN regulating AD, OPN binds to downstream receptor CD44 to further exert neuroprotective and remodeling activities. Due to the important role of the OPN–CD44 complex in neuroprotection and remodeling, enhancing OPN expression can inhibit neuro-damaging phenomena in AD [53,54]. Exercise stimulates OPN secretion and increases its expression levels in the bone. Strengthening OPN expression can better inhibit neuro damage in AD. In the Aβ clearance mechanism of AD, OPN can also regulate macrophage immune resistance to Aβ deposition. OPN promotes the phagocytosis of Aβ fibrils and related receptors, changes cell morphology, reduces inducible nitric oxide synthase (iNOS) levels, and enhances the anti-inflammatory effects of interleukin-10(IL-10) and matrix metalloproteinase 9 (MMP-9) [47] (Figure 1).

## 3. Exercise-Induced Modulation of Muscle Factors Improves Alzheimer’s Disease Symptoms through “Muscle–Brain Crosstalk”

Exercise can regulate the expression of various myokines such as brain-derived neurotrophic factor (BDNF), irisin, interleukin-6 (IL-6), cathepsin B (CTSB), myostatin (MSTN), insulin-like growth factor-1 (IGF-1), and vascular endothelial growth factor (VEGF) released from active skeletal muscle tissues. These exercise-induced myokines enter the circulation and are able to cross the blood–brain barrier. This establishes a communication pathway between the muscles and the brain, known as the “muscle–brain crosstalk” mechanism. Through this mechanism, myokines can influence important brain functions. Specifically, they are involved in maintaining neuronal structure and health, enhancing synaptic plasticity, reducing neuroinflammation, and promoting neurogenesis as well as cerebral blood vessel formation. This occurs as the myokines interact with neurons and neural cells in the brain. The muscle–brain crosstalk mechanism provides an explanation for how physical activity can positively impact cognitive functions. It describes the role of contracting skeletal muscles as an endocrine organ that secretes myokines to directly influence and regulate brain processes. This communication between tissues improves neurological outcomes related to learning, memory, mood, and neurodegenerative diseases.

1. BDNF, a neurotrophic factor, plays a crucial role in neurogenesis and synaptic plasticity. Its low levels are linked to AD, with studies indicating reduced BDNF in AD patients and animal models [55,56,57]. BDNF can cross the blood–brain barrier, enhancing neurotrophic production in the hippocampus and supporting cognitive function [58,59]. Exercise has been shown to increase BDNF secretion in muscle tissue, correlating with exercise intensity and leading to cognitive improvements in both humans and animal models of AD [60,61,62]. Long-term exercise can elevate baseline BDNF levels, with evidence of hippocampal growth and better spatial memory after a year of aerobic exercise [63,64]. BDNF acts through the TrkB receptor, activating pathways like MAPK and PI3K, which are important for neuronal survival and plasticity [65]. It also modulates amyloid-beta (Aβ) production by enhancing alpha-secretase activity and reducing BACE1 levels, mitigating Aβ-induced toxicity [66,67,68,69,70]. Thus, exercise-induced BDNF not only offers direct neuroprotective effects but may also contribute to the reduction in AD pathology. These findings illustrate that BDNF plays a dual role in maintaining brain health and combating AD. By promoting neurogenesis and modulating pathological processes associated with AD, BDNF emerges as a promising therapeutic target. The research underscores the importance of long-term exercise, not only for its direct elevation of BDNF levels but also for its potential benefits in cognitive enhancement and the deceleration of AD pathology. Future studies may focus on how to maximize the impact of exercise on BDNF and how to translate these findings into concrete preventative and therapeutic strategies.
2. Irisin, a myokine released during exercise, is produced by the cleavage of FNDC5 and affects energy metabolism and neuroprotection [71]. It promotes the browning of white adipose tissue, enhances insulin sensitivity, and is implicated in the pathophysiology of AD, with lower levels observed in AD models [62,72,73]. Exercise activates PGC-1α, up-regulating FNDC5/Irisin and reducing amyloid-beta (Aβ) production [74,75]. Irisin levels rise in response to exercise, potentially improving cognitive functions via various mechanisms, including promoting neuronal growth and reducing inflammation [76,77,78,79,80,81,82,83,84,85,86,87,88,89]. Irisin also influences the production of BDNF, a factor crucial for neuronal health [90,91,92]. Considering AD’s metabolic aspects, often termed “type 3 diabetes,” Irisin’s role in energy homeostasis and insulin sensitivity is of particular interest, offering a potential therapeutic avenue for AD and diabetes [93,94,95,96]. In summary, Irisin emerges as a promising molecule linking exercise to metabolic and cognitive health. Its multifaceted role in energy regulation, neuroprotection, and potential to mitigate AD symptoms underscores the need for further research. Future studies should focus on the therapeutic potential of Irisin, aiming to harness its benefits for treating metabolic and neurodegenerative disorders in aging populations.
3. IL-6, a cytokine released from muscles during exercise, plays complex roles in the body and is linked to AD. High baseline levels of IL-6 are associated with increased risk of AD and cognitive decline in the elderly [97,98,99,100,101]. This cytokine has dual effects in AD: it can exacerbate neuronal damage by enhancing APP synthesis and Aβ toxicity, yet it also supports neurogenesis and gliogenesis through specific signaling pathways [102,103,104]. Acute exercise raises IL-6 levels, which may help regulate inflammation and promote neuroprotective responses [105,106]. Chronic exercise, conversely, is associated with reduced resting IL-6 levels and may prevent its detrimental effects in the brain [107,108,109]. In essence, while acute exercise triggers a beneficial IL-6 response that protects the brain, chronic exercise lowers the baseline of IL-6, potentially reducing chronic inflammation and the risk of neurodegenerative diseases. This suggests that exercise, both acute and chronic, can be a strategic approach to modulate IL-6 levels for brain health, emphasizing the importance of physical activity in the prevention and management of AD.
4. IGF-1 plays a crucial role in CNS function by enhancing synaptic plasticity and density, with a decline in IGF-1 associated with AD cognitive symptoms [110,111]. Exercise-induced IGF-1 secretion from muscles benefits brain health, potentially reducing AD pathology through mechanisms like inhibiting Aβ production and tau phosphorylation via the IRS1/PI3K/Akt/mTOR pathway [112]. It also supports hippocampal neurogenesis and BDNF regulation, integral to neuronal growth and differentiation [113,114,115]. Similarly, VEGF, a key angiogenesis regulator, promotes vascular and neural health. Its overexpression in rodent models boosts hippocampal angiogenesis and neurogenesis, while its inhibition can negate exercise-induced neurogenic benefits, highlighting the importance of muscle–brain crosstalk [116,117,118,119]. These studies highlight how exercise promotes brain health by stimulating the secretion of molecules such as IGF-1 and VEGF, particularly with respect to neuroprotection and neurogenesis associated with AD. These findings reveal the biochemical links between physical activity and brain health, offering potential strategies for the prevention and treatment of AD. By enhancing the health of the vascular and nervous systems, exercise not only aids in improving cognitive functions but may also slow the progression of AD (Figure 1).

## 4. Exercise Regulates the Gut Microbiota and Improves Symptoms of Alzheimer’s Disease through “Gut–Brain Crosstalk”

As humans evolved, the gut microbiota co-evolved, leading to the development of complex immune mechanisms that monitor and regulate the gut ecosystem. The crosstalk between the gut microbiota, gut, and brain plays a vital role in maintaining health and various diseases [120,121]. The gut microbiota-gut–brain axis serves as a bidirectional regulatory axis between the central nervous system and the gut microbiota. There are several key mechanisms through which the gut–brain axis facilitates crosstalk: Neurological pathways—The gut microbiota can influence the gut nervous system, regulating gut motility and permeability, which subsequently impacts the hypothalamic–pituitary–adrenal axis and autonomic nervous system, affecting mood and cognition [122,123,124,125]. Immune pathways—Activity of the gut microbiota can influence the gut immune system and cytokine secretion, with cytokines then able to access the brain by crossing the blood–brain barrier. Neurotransmitter pathways—Gut microbiota activity can influence levels of neurotransmitters like serotonin both centrally and in the gut. Metabolic pathways—Small molecules produced by gut microbiota metabolism, such as short-chain fatty acids, can directly or indirectly influence neural activity in the brain through blood circulation. These pathways constitute the gut microbiota-gut–brain axis, establishing the physiological basis for gut–brain crosstalk. Exercise, as a non-pharmacological therapy for preventing and mitigating the progression of AD, not only regulates the gut microbiota but also acts on the neuroimmune pathways within the gut–brain axis, suppressing brain/peripheral inflammation induced by the gut microbiota and improving multiple aspects of AD pathogenesis [126,127].

Long-term, moderate-intensity exercise has been shown to regulate brain Aβ generation and clearance, with additional benefits including gut microbiota remodeling [128,129]. Exercise alters the abundance of various microbial taxa, which may in turn affect Aβ levels and cognitive functions in AD mice [130,131,132]. High-intensity interval training also shows promise in modulating the gut microbiota and enhancing the relative abundance of beneficial bacterial species [131].

Exercise impacts the gut microbiota–immune–neural pathway, potentially alleviating inflammation associated with AD [133]. It regulates metabolic products like kynurenine and short-chain fatty acids (SCFAs), influencing brain and peripheral inflammation [134,135,136,137,138,139]. Exercise-induced changes in the gut microbiota can also influence microglial cell maturation and function, which is crucial for the innate immune response in the central nervous system [140,141,142].

Different exercise intensities and modalities may differentially affect SCFA levels, with implications for microglial phagocytosis and inflammation [143,144,145]. Exercise can promote a shift from pro-inflammatory M1 to anti-inflammatory M2 microglia, contributing to reduced brain inflammation [146,147]. Additionally, exercise can enhance gut immune function, evidenced by increased IgA levels and altered cytokine expression [148], and modulate peripheral inflammation through muscle and hypothalamic–pituitary–adrenal axis pathways [149,150,151]. A 12-week exercise program has been shown to increase gut microbiota diversity, improve gut barrier integrity, and reduce gut and brain inflammation in rats, highlighting its potential to mitigate AD pathology [152].

In summary, exercise has a multifaceted role in influencing gut microbiota composition, immune response, and inflammation, all of which are important factors in the pathogenesis of AD. Different types and intensities of exercise can modulate levels of SCFAs and other metabolic products, which in turn can affect immune cell function and inflammatory responses in the brain and periphery. Exercise promotes a shift towards anti-inflammatory microglial profiles and enhances gut immune function, potentially protecting against AD progression. By improving gut microbiota diversity and barrier function, exercise may offer a non-pharmacological approach to managing AD-related inflammation and cognitive decline (Figure 1).

## 5. Effects of Different Exercise Intervention Programs on Symptom Improvement in Alzheimer’s Disease

### 5.1. Effects of Different Types of Exercise on Symptom Improvement in Alzheimer’s Disease

#### 5.1.1. Aerobic Exercise

Walking exercise is a widely accepted and favored physical activity among middle-aged and elderly individuals. A study by Friedman, R. et al. [153] revealed that a 10-week program of walking, conducted three times per week for 30 min per session, demonstrated improvements in the communication abilities of individuals with moderate to severe AD. Venturelli et al. [154] conducted a 24-week intervention involving walking exercises at least four times per week for 30 min per session in late-stage AD patients residing in nursing homes, resulting in enhanced 6 min walk test scores, activities of daily living, and a slowed decline in Minimum Mental State Examination (MMSE) scores. Arcoverde et al. [155] implemented a 4-week adaptation period on a treadmill for eight AD patients, gradually increasing intensity and duration until reaching 40% of their maximal oxygen uptake (initial speed: 2 km/h, initial duration: 20 min). Following the adaptation period, patients engaged in treadmill walking twice a week for 30 min per session for a duration of 3 months, resulting in improved cognitive function. Additionally, sleep disturbances are common among AD patients [156]. Research indicated that daily walking for 30 min improved sleep efficiency and quality in community-dwelling AD patients [157].

Cycling exercise on a stationary bike is a form of non-weight-bearing aerobic training with minimal risk of falling. A study by Yang et al. [158] divided mild AD patients into a cycling exercise group and a control group. The cycling exercise group performed moderate-intensity cycling for 40 min, three times per week, over a period of three months. The results showed that moderate-intensity cycling exercise can improve cognitive function in individuals with mild AD. Similarly, regular moderate-intensity running training for three months in AD patients can also enhance cognitive function. AD patients exhibit increased absorption of ketone bodies by the body, with higher plasma concentrations of acetoacetate and enhanced blood–brain acetoacetate influx rate. These findings suggest that maintaining long-term regular aerobic exercise can increase the brain’s glucose absorption and promote brain energy metabolism. For individuals with mild AD, a 16-week exercise program consisting of moderate to high-intensity aerobic exercise on a treadmill and stationary bike, three times per week for one hour per session, can improve cognitive function [159]. Another study found that a 16-week aerobic exercise program, consisting of three sessions of 60 min per week, improved cognitive function, quality of life, and daily activity abilities in individuals with mild AD [160]. Older adults with mild to moderate AD dementia who participated in a six-month program of moderate-intensity cycling and stretching exercises, three times per week for 20–50 min, showed a potential reduction in cognitive decline compared to individuals with mild-to-moderate Alzheimer’s disease. However, aerobic exercise did not demonstrate superior cognitive effects compared to stretching [161].

There is a wide variety of aerobic exercises that can be chosen by individuals with AD. Considering that older adults are at a higher risk of developing AD, selecting the most appropriate exercise type based on the individual’s specific circumstances is crucial. Walking is considered as a favorable option for elderly patients without regular exercise habits. For individuals with mild AD, opting for exercise modalities that are engaging and interactive can contribute positively to their overall physical and mental health.

However, some studies have not observed positive effects of exercise interventions on cognitive function in AD patients. Cott et al. [162] conducted a 16-week aerobic exercise training on AD patients, but no significant improvement in cognitive function was observed. Hoffmann et al. [160] had 190 AD patients engage in moderate to high-intensity aerobic exercise, but no significant improvement in cognitive function was found compared to the control group. Although aerobic exercise has shown significant impact on improving cognitive function in AD patients, more analytical research and evaluation are needed regarding the intervention effects of exercise on cognitive function in AD patients (Table 1).

#### 5.1.2. Resistance Exercise

A meta-analysis conducted by Lv et al. [169] included 24 studies on different exercise interventions for cognitive function in AD patients. The results showed that various exercise interventions had beneficial effects on overall cognitive function, with resistance training being the most effective (72.4%) exercise modality for improving cognitive function in AD patients (including aerobic exercise, resistance exercise, and multi-component exercise). In a study by Ahn [163], elastic band resistance training was conducted on 23 patients with mild AD, with three sessions of upper and lower limb exercises per week for 5 months. Measurements of physical health were assessed using chair leg squat, single-leg stance, timed up-and-go test, 2 min walk test, and gait ability before and after the exercise intervention. The study found improvements in muscle strength and endurance, as well as cardiovascular function and speed in the patients. Portugal et al. [170] also conducted a similar study and found that long-term resistance training could reduce oxidative stress, enhance serum concentrations of BDNF and insulin-like growth factor I, and improve cognitive abilities. Based on these results, they hypothesized that resistance training may be associated with increased neurogenesis and neural plasticity, thereby counteracting the effects of brain aging. To further support the notion that resistance training can improve AD, Liu et al. [171] performed resistance ladder training on 9-month-old mice with cognitive impairment. The results showed that compared to the sedentary group, resistance training improved cognitive abilities in the frontal cortex and hippocampus of the mice. Additionally, assessments revealed that resistance training had positive effects on neuroinflammation, amyloid-beta and tau pathology, and synaptic plasticity. Similar findings were reported by Tsai et al. [172], who observed improvements in AD symptoms and reduced inflammatory factors. Their study, which involved 55 patients with mild cognitive impairment over a 16-week period, found that the resistance exercise group showed a significant increase in the levels of the circulating neuroprotective growth factor IGF-1, as well as a decrease in interleukin-15 levels.

Research has shown that resistance exercise twice a week can improve brain plasticity, which may be positively correlated with better performance in executive functions [173]. Furthermore, resistance exercise can increase muscle strength, control, and coordination, which also have a positive impact on executive functions [174]. Among cognitive functions, memory function is least easily influenced by exercise [175]. This may be due to resistance exercise increasing blood flow to brain regions associated with memory [176]. Resistance exercise should receive more attention in clinical rehabilitation programs as it can significantly enhance overall cognitive and memory function in Alzheimer’s disease patients, alleviating their decline (Table 1).

#### 5.1.3. Multimodal Exercises Combination

The combined intervention of multiple types of exercise for patients with AD has received increasing attention. The decline in cognitive abilities in AD patients is caused by multiple factors, and the impact of single exercise or cognitive interventions on cognitive functions of patients is relatively small. In contrast, the combination of exercise and cognitive intervention is more effective, as they can interact with each other. In patients with mild cognitive impairment, compared to single interventions, the use of exercise combined with cognitive training can improve their cognitive function and activities of daily living in a shorter period of time. Exercise-based gaming, as a form of combined exercise and cognitive training, is a comprehensive intervention that combines physical exercise with interactive virtual reality. For example, interactive physical–cognitive training combines stationary cycling with virtual reality tourism, providing patients with cycling exercises and cognitive stimulation. It helps improve motor control, enhance frontal lobe cognitive function and neuroplasticity, and significantly improve overall cognition (working memory, episodic memory, and executive function) [177].

A recent network meta-analysis compared the impact of aerobic exercise, resistance exercise, and combined multimodal exercise on cognitive function in patients with AD. The findings revealed that combined multimodal exercise was the most effective approach for enhancing executive function (30.4%). According to the authors, combined multimodal exercise outperformed aerobic exercise and resistance exercise in terms of promoting executive function [169]. Furthermore, there is substantial evidence suggesting that individuals aged 50 and older should engage in aerobic and resistance exercise to improve brain health [178]. The mechanisms underlying the improvements in executive function observed with combined multimodal exercise may be associated with various exercise tasks such as perception–movement adaptation and variable fiber bundle coordination. Future research could comprehensively examine the neurophysiological mechanisms associated with different exercise modalities’ ability to enhance executive function, thereby elucidating their impacts on structural and functional brain plasticity. In a study [164] involving patients with moderate AD (69 years old), short-term moderate-intensity aerobic exercise (20 min of cycling at 60% of maximum heart rate) combined with cognitive games resulted in improved cognitive function and increased functional activity time. Following a six-month training regimen (comprising aerobic exercise, endurance training, flexibility exercises, and postural exercises), AD patients (84 years old) showed enhanced physical and cognitive function. Exercise sessions lasted 45–55 min, twice a week [165].

Another systematic review examined the effects of physical exercise on functional capacity improvement in older adults with Alzheimer’s disease. The review indicated that a multimodal exercise program (conducted at home, twice a week, for 75 min per session, over three months) could enhance functional capacity (activities of daily living) among individuals with AD. The multimodal exercise program primarily involved aerobic, balance, muscle, and flexibility training [179]. A 12-month multicenter randomized controlled trial by Rolland et al. [166] divided AD patients residing in nursing homes into an exercise group and a usual care group. The exercise group underwent various forms of exercise, including aerobic exercise, strength training, flexibility exercises, and balance training, twice a week for one hour per session. The study’s findings demonstrated that the exercise group exhibited about a one-third decrease in the Activities of Daily Living (ADL) scale score compared to the routine medical care group [166]. The researchers believed that different types of exercise appeared to have a protective effect on Alzheimer’s disease by reducing oxidative stress, lowering Aβ levels, increasing antioxidant systems, and promoting brain flexibility [180] (Table 1).

### 5.2. Effects of Different Exercise Intensities and Frequencies on Symptom Improvement in Alzheimer’s Disease

The impact of different exercise intensities, frequencies, and modes on improving symptoms of Alzheimer’s disease varies. A meta-analysis study involving 36 randomized controlled trials with adults aged 50 and above was conducted to investigate the effects of physical exercise on cognitive function [181]. The findings revealed that exercise had a significant positive influence on cognitive function, irrespective of participants’ baseline cognitive status. However, exercise intensity was found to play a crucial role in modulating the beneficial effects of exercise on cognition, with only high-intensity and moderate-intensity exercise training showing significant impacts [181]. Another randomized controlled trial focused on sedentary older adults, examining the effects of 12 weeks of high-intensity interval training, moderate-intensity continuous training, and stretching exercises. The results emphasized the correlation between exercise intensity, improvements in cardiorespiratory function, and cognitive benefits. Specifically, the high-intensity training group demonstrated noteworthy enhancements in memory performance. Moreover, overall improvements in cardiorespiratory function were positively associated with memory test performance [113].

A study evaluating different exercise intensities and forms in cognitive function improvement among AD model mice revealed that high-intensity running training yielded superior results for cognitive function improvement in Tg2576 mice compared to the low-intensity group. Similarly, medium-intensity running training exhibited a more significant inhibitory effect on Aβ formation in 2×Tg-AD mice compared to the low-intensity group. These findings suggest that moderate- to high-intensity exercise training has a greater impact on enhancing cognitive function [182,183]. Moderate- to high-intensity exercise primarily enhances cognitive function by stimulating the release of antioxidant enzymes and neurotrophic factors such as BDNF, IGF-1, and VEGF. Conversely, low- to moderate-intensity exercise mainly reduces the highly phosphorylated levels of intracellular Tau protein and the quantity of extracellular Aβ plaques, thereby alleviating neuroinflammation and slowing down AD progression [184]. High-intensity exercise may be a suitable exercise type for middle-aged and older individuals to reduce the risk of AD. This form of exercise potentially induces higher lactate levels, consequently leading to a greater increase in BDNF, which is beneficial for cognitive function [185,186].

In addition, high-frequency exercise has a greater cognitive intervention effect on individuals with AD compared to low-frequency exercise, and high-frequency exercise results in better improvement of cognitive function in AD patients. The increase in exercise volume may be attributed to the increased exercise volume brought about by high-frequency exercise. Studies have found that within a certain range, the larger the exercise volume, the more significant the improvement in cognitive function in dementia patients. Exercise can increase blood supply to brain tissues and improve cognitive function in dementia patients, and this benefit is positively correlated with exercise volume [187]. Under the same intensity, the same exercise mode, and the same level of dementia, only long-term exercise interventions (over 24 weeks) can significantly improve spatial memory, while short-term exercise (less than 12 weeks) only partially improves the mental state of patients, indicating that continuous exercise intervention is needed to improve cognitive function [188]. There is a threshold for the impact of exercise intervention on cognitive effects. If the exercise intensity is lower than the threshold, cognitive function cannot be effectively improved [188]. For dementia patients at different levels, the same intensity of exercise will also have different effects. For example, moderate-intensity exercise can improve spatial memory in patients with cognitive impairment but can only delay the decline in cognitive level in AD patients. The duration of exercise also has an impact on the treatment outcome for the same type of patients. In AD patients, 2 h of walking per week has a more significant effect on improving daily living abilities and reducing neurological and psychiatric symptoms compared to 1 h of walking, and it can also improve mini-mental state examination (MMSE) scores [188].

Animal experiments and human trials have shown that different forms of exercise, intensities, and frequencies have varying effects on cognitive function improvement. Therefore, personalized exercise strategies need to be developed based on factors such as the patient’s cognitive level, physical fitness, interests, and environmental conditions. However, there is still controversy regarding the cognitive effects of exercise interventions, with some suggesting that exercise interventions have either no or limited impact on cognition [167,168]. A key reason for this cognitive discrepancy is the diversity of evaluation methods, without the use of standardized assessment scales. The general consensus is that exercise can enhance cognitive, learning, and memory abilities in AD patients, as well as synaptic plasticity and neuroprotective effects (Table 1).

### 5.3. Characteristics and Risk of Bias in Exercise Intervention Studies for Alzheimer’s Disease

In evaluating the robustness of the extant literature, the risk of bias emerges as a pivotal factor in ascertaining the reliability and validity of the reported findings. A recurrent limitation across the studies under review is the prevalence of small sample sizes, which amplifies the potential for random errors and undermines the stability and generalizability of the results.

Furthermore, the majority of the studies fail to provide comprehensive details regarding the implementation of blinding procedures or other bias mitigation strategies. The absence of such methodological rigor can lead to performance and detection biases, skewing the interpretation of intervention efficacy.

Additionally, the application of statistical methods in some studies appears either inadequately explained or inappropriate, casting doubt on the validity of the analytical outcomes. The selection and deployment of statistical techniques must align with the nature of the data and the design of the study to ensure the integrity of the findings.

The heterogeneity and low consistency of results across multiple studies suggest potential outcome instability or the influence of unaccounted confounding factors.

Notwithstanding these concerns, a subset of studies demonstrates a commitment to methodological rigor, employing larger sample sizes and sophisticated statistical models that bolster the credibility of their findings. Yet, even within this context, the opaque reporting on blinding and bias reduction measures persists as a concern.

To conclude, while the body of research offers preliminary evidence on the effects of exercise interventions on Alzheimer’s disease, caution is warranted in the interpretation of these findings. Future investigations would benefit from larger cohorts, enhanced methodological stringency, transparent reporting of methodologies, and consistent outcomes to elevate the quality of research and enhance the reliability of the evidence presented (Table 2).

## 6. The Role of Artificial Intelligence and Neuroimaging Technologies in Exercise Intervention for Alzheimer’s Disease

In the diagnosis and treatment of AD, artificial intelligence and neuroimaging techniques have played important roles. Artificial intelligence technologies such as machine learning and deep learning can analyze a large amount of patient data to help doctors accurately diagnose AD, predict disease progression, and provide support for personalized treatment plans. James et al. [189] used four machine learning algorithms to establish an AD prediction model based on 15,307 subjects, and found that only six sets of patient information, including MMSE score, completion time of line drawing test, orientation, memory, household and hobby scores in the Clinical Dementia Rating Scale (CDR), and independence score, can be used to predict the risk of AD in the next two years with 91% accuracy and reduce misdiagnosis rate by 84%. Ezzati et al. [190] evaluated the accuracy of using machine learning methods combined with the ATN biomarker classification system to predict the progression of MCI to dementia in 415 MCI patients in the AD Neuroimaging Initiative (ADNI) database, demonstrating that machine learning methods have better diagnostic efficiency than empirical assessment. Hammond et al. [191] extracted AD-related biomarkers from the ADNI database and evaluated the efficacy of each indicator in predicting the clinical status of normal cognition, MCI, and AD using the random forest machine learning algorithm, among which Aβ and phosphorylated tau protein had higher contributions in predicting early cognitive impairment, and glucose uptake predicted late cognitive impairment better. These results can guide clinicians to make relevant management decisions based on the disease staging of ATN biomarkers and provide a theoretical basis for drug development teams to design corresponding treatment methods according to different pathological and physiological changes in the disease. At the same time, neuroimaging techniques such as functional magnetic resonance imaging (fMRI) and electroencephalography (EEG) can observe changes in brain function in patients, help doctors better understand the development trend of the disease, evaluate the intervention effect, and provide fine guidance in treatment. Arterial spin labeling (ASL) can use magnetically labeled arterial blood as an endogenous contrast agent to quantitatively measure microvascular attention in the brain. Studies have found that regions such as the frontal lobe, temporal lobe, and hippocampus of MCI and AD patients have reduced blood flow compared to normal controls, and perfusion of the frontal lobe is most sensitive to the prediction of future cognition [192]. Researchers have evaluated CBF changes in AD patients undergoing therapeutic exercise using MRI sequences with pulsed arterial spin labeling (PASL) and concluded that exercise may have no effect on CBF in mild to moderate AD patients [193]. fMRI imaging of the brain during cognitive exercises can show functional improvement of cognitive and cortical networks related to exercise or health. In this regard, some randomized clinical trials targeting young adults showed that aerobic exercise increased connectivity and cortical activity compared to control groups [194,195]. The application of these artificial intelligence and neuroimaging techniques provides more accurate and effective means for the diagnosis and treatment of Alzheimer’s disease.

In the intervention of other diseases, artificial intelligence and neuroimaging techniques have exhibited promising and successful applications. For example, artificial intelligence has made significant breakthroughs in the field of lung cancer. Researchers have developed methods to automatically generate lung cancer radiation therapy plans using deep learning networks and a vast amount of lung imaging data. By analyzing imaging data, pathological features, and prognostic factors, this method can accurately predict tumor response and patient survival rates, providing more precise treatment strategies for clinical practice [196,197,198]. Furthermore, neuroimaging techniques play a crucial role in stroke intervention. Non-invasive technologies such as functional magnetic resonance imaging (fMRI) and electroencephalography (EEG) allow for the non-invasive observation of patients’ brain activity, aiding in the evaluation of functional recovery and brain reorganization, which guides rehabilitation therapy and intervention planning. Research indicates that by combining neuroimaging techniques and machine learning algorithms, it is possible to predict stroke patients’ recovery process and prognosis, enabling the customization of rehabilitation programs and improving rehabilitation outcomes [199,200,201,202].

The successful applications mentioned above provide inspiration for exploring and utilizing artificial intelligence and neuroimaging techniques in the exercise intervention for AD. By employing similar methods and technologies, artificial intelligence algorithms can be employed to analyze exercise intervention data, provide insights into changes in motor ability for Alzheimer’s patients, predict disease progression, and support the development of personalized intervention plans for doctors. Meanwhile, in conjunction with neuroimaging, changes in brain function can be observed, with potential to better understand the correlation between exercise intervention and its effects, in order to guide optimization and modification of exercise intervention plans. Such exploration and implementation efforts have the potential to provide more effective and personalized exercise intervention strategies for Alzheimer’s patients.

The importance of exercise intervention in AD is well recognized, with evidence suggesting that moderate physical activity can enhance cognitive function and potentially slow disease progression. The use of artificial intelligence (AI) and neuroimaging techniques can greatly refine the approach to exercise interventions by offering personalized plans tailored to the unique characteristics of each patient. AI algorithms can analyze extensive data to identify individual risk factors and physical capabilities, informing the development of customized exercise regimens.

Neuroimaging can track changes in brain function, providing insights into how exercise affects the brain and cognition in AD patients. This data can guide doctors in fine-tuning exercise programs to maximize cognitive benefits. Wearable sensors and smart devices can monitor patients’ physical activity and condition in real-time, offering immediate feedback. AI can process this data to give patients and clinicians actionable advice and adapt interventions as needed.

However, the application of AI and neuroimaging in AD exercise interventions comes with challenges. The progressive nature of AD means that patients’ abilities can change, requiring continuous adaptation of exercise plans. AI systems require large datasets to learn from, and there are concerns about data privacy and the ethical use of personal health information. Neuroimaging techniques, while valuable, are expensive and not universally accessible, and repeated exposure to certain imaging modalities may not be advisable.

Furthermore, ensuring patient engagement and adherence to exercise programs over time is a significant hurdle. Motivation can be difficult to maintain, especially as the disease progresses and cognitive and physical functions decline. The interpretation of data from AI and neuroimaging can be complex, and clinicians must be trained to understand and apply this information effectively.

In summary, while AI and neuroimaging offer exciting possibilities for enhancing exercise interventions in AD, their integration into clinical practice must be managed carefully. These technologies must be used ethically and with consideration for the limitations they present.

## 7. Discussion

This review explores how exercise interventions may mitigate cognitive decline and neurodegeneration in AD through neurobiological mechanisms. Exercise benefits extend beyond physical health, also impacting cognitive function and brain health by facilitating complex interactions among the body’s circulatory, immune, and nervous systems. Investigating these interactions may illuminate new therapeutic strategies for AD. Exercise stimulates “cross-talk” between the body and brain. The skeleton, as an endocrine organ, secretes factors like SOST and OCN that influence brain physiology and metabolism in AD, with exercise enhancing this “bone–brain cross-talk”. Similarly, skeletal muscle secretes myokines that modulate brain function, with exercise altering their expression to inhibit β-amyloid deposition and improve central nervous system structure and function, known as “muscle–brain cross-talk”. The gut microbiota–brain axis also plays a role in AD progression. Exercise can improve this axis by modifying gut microbiota and reducing Aβ and inflammation, offering a multi-target, low-risk intervention for AD. Different exercise types have unique benefits for AD. Aerobic exercise boosts neurogenesis and executive function, while resistance training enhances brain plasticity. Multimodal training may benefit brain structure and function through diverse sensory-motor tasks. Personalized exercise regimens considering cognitive level, physical fitness, and preferences are recommended, with moderate-intensity, high-frequency exercise showing the most promise. Advancements in artificial intelligence and neuroimaging could further refine exercise interventions for AD, providing customized plans and real-time patient feedback, thereby improving patient outcomes and quality of life.

This review highlights the potential of exercise in slowing AD progression, suggesting several research avenues. Future studies could focus on optimizing exercise variables such as duration, intensity, and frequency for AD patients, and comparing the efficacy of different exercise types, including aerobic, resistance, and mind–body exercises like yoga. Long-term effects of exercise are also critical, necessitating longitudinal studies to evaluate the sustained benefits on cognitive decline, functionality, and quality of life in AD. Interdisciplinary efforts among exercise science, neurology, and gerontology are essential for holistic insights into exercise and AD. Personalized exercise interventions, considering individual needs and disease stages, are the ultimate aim for prevention or treatment strategies. Challenges include tailoring exercise for varying AD severities and ensuring patient compliance, potentially through technology like video games, virtual reality, AI, and wearable devices. Involving caregivers can also improve adherence. Progress in this field could significantly impact AD prevalence and patient quality of life.

A healthy lifestyle, particularly regular physical activity, is crucial in preventing the transition of β-amyloid protein (Aβ) from its physiological state to a pathological state [203]. Previous studies have shown that metabolic abnormalities and cardiovascular risk factors may promote the pathological aggregation of Aβ, which is a critical step in the pathogenesis of Alzheimer’s disease [204]. Thus, by counteracting these risk factors, regular physical activity may play a role in preventing the pathological activity of Aβ. Specifically, physical activity reduces the risk of cardiovascular disease through multiple mechanisms, including lowering blood pressure, improving cholesterol levels, increasing insulin sensitivity, and reducing inflammation. Moreover, exercise enhances cerebral blood flow, improves neural plasticity, and promotes the release of neurotrophic factors, all of which may help maintain Aβ at a physiological level and prevent its excessive aggregation into a pathological form. Therefore, incorporating physical activity as a part of daily life is not only crucial for overall health maintenance but also potentially an effective strategy to prevent or delay neurodegenerative diseases such as Alzheimer’s disease.

The review offers significant practical implications for healthcare providers and caregivers of patients with AD. By elucidating the neurobiological mechanisms through which exercise may confer its benefits, it provides a scientific foundation for the integration of exercise into therapeutic plans. It emphasizes that exercise has the potential to not only improve cognitive functions and slow the progression of the disease but also to enhance the quality of life for patients by improving mood and reducing behavioral symptoms. Additionally, the review compiles evidence on the efficacy of various exercise modalities and intensities, guiding medical professionals in creating personalized and beneficial exercise programs for AD patients at different stages of the disease. The practical significance is further highlighted by discussing methods to assess and monitor the effectiveness of exercise interventions, ensuring that caregivers and healthcare providers can evaluate progress and adjust interventions as necessary. The review underscores exercise as a valuable non-pharmacological strategy that supplements existing pharmacological treatments, advocating for a more comprehensive approach to AD management that can be easily implemented in both clinical and home-care settings. These insights are crucial in equipping medical practitioners and caregivers with the knowledge and tools necessary to optimize the care and support provided to individuals living with Alzheimer’s disease.

In exploring exercise interventions for AD patients and assessing their neurobiological mechanisms and effects, we must acknowledge the inconsistencies present in the literature. These discrepancies may stem from a variety of factors, including differences in study design, limitations in sample size, diversity in the type and intensity of exercise interventions, and the varied methods and timing of outcome assessments. To gain a clearer understanding of the impact of exercise on AD patients, future research should employ more standardized and systematic approaches to ensure comparability and replicability of results. When considering potential risks, although exercise is generally viewed as a low-risk activity beneficial to health, it may require more cautious consideration for AD patients, particularly those in the later stages of the disease or with other comorbidities. Possible risks include falls, cardiovascular events, and excessive fatigue. Therefore, exercise programs should be conducted under the supervision of professionals and tailored to the specific health status of each patient. For different AD patient groups, it is essential to recognize that the stage of AD progression, age, gender, physical capabilities, and quality of life may all affect the outcome of exercise interventions. For instance, patients in the early stages of AD may be able to engage in more varied and intense exercises, while those in later stages might be better suited for low-intensity, low-risk activities such as balance and flexibility training. Moreover, exercise plans should take into account the patients’ personal preferences and previous exercise experiences to enhance participation and sustainability. By considering these factors comprehensively, we can design exercise interventions in a more targeted manner to maximize their positive impact on the cognitive and physiological health of AD patients.

This review has some limitations. One limitation is its reliance on existing literature, which may be influenced by publication bias and selective reporting of results. Additionally, more randomized controlled trials are needed to rigorously assess the impact of exercise on AD. The studies reviewed in this analysis were mostly observational or small-scale trials, and larger, more rigorous studies are needed to confirm these findings and establish causality. Despite these limitations, this review has several important strengths and value. The analysis provides an overview of the current knowledge about the relationship between exercise and AD, including research on the beneficial mechanisms of exercise on cognitive function and a critical evaluation of the existing evidence. The review also highlights the potential role of artificial intelligence and neuroimaging techniques in exercise interventions for AD. Overall, this review offers valuable insights into the potential benefits of exercise for cognitive health. The findings of this review have important implications for clinical practice. Exercise is a potentially accessible and cost-effective intervention that can improve cognitive function and potentially delay the onset or progression of AD. However, further validation of these findings through clinical trials is needed, along with guidance on the optimal type, intensity, and duration of exercise for different populations. Additionally, incorporating exercise interventions into standard care protocols will require collaboration among healthcare providers, physical therapists, and community organizations. In conclusion, the potential translation of the findings of this review into clinical practice underscores the importance of continued research in this field and the need to identify effective strategies for promoting physical activity among older adults.

## 8. Conclusions

Exercise brings many benefits and may influence Alzheimer’s disease through different pathways, particularly through the connections between skeletal system and brain, muscular system and brain, and gut–brain axis. Through these crosstalk mechanisms, exercise interventions may help improve the progression of Alzheimer’s disease, with moderate-intensity, multimodal exercise showing better outcomes. However, despite various mechanisms proposed in preclinical studies as potential mediators of exercise benefits in Alzheimer’s disease, there still a lack of evidence from human trials. Therefore, further research is needed to elucidate the beneficial crosstalk mechanisms induced by exercise, validate the applicability of these benefits to elderly individuals with Alzheimer’s disease, and determine the most effective intervention strategies. Future research should involve long-term follow-up studies to track the progression of Alzheimer’s disease in individuals undergoing exercise interventions. These studies must establish a connection between changes in exercise regimens and alterations in cognitive function as well as neuroanatomical structures, incorporating artificial intelligence and neuroimaging technologies. The goal is to develop personalized and effective exercise interventions aimed at enhancing the quality of life for patients and their families. By longitudinally monitoring individuals and associating exercise programs with cognitive and neural changes, we can gain a clearer understanding of the mechanisms by which exercise may modify the course of Alzheimer’s disease. The application of advanced technologies will aid researchers in validating tailored exercise prescriptions that meet the unique needs of each patient, thereby optimizing treatment outcomes and improving quality of life for those affected by this debilitating condition.

## Figures and Tables

**Table 1:** Exercise interventions and their effects on Alzheimer’s disease outcomes.

Author (s)	Exercise Type	Intensity	Frequency	Outcomes
Friedman, R. et al., 1991 [153]	walking	Low	3 times/week × 30 min × 10 weeks	communication performance improved
Venturelli, M. et al., 2011 [154]	walking	Moderate	4 times/week × 30 min × 24 weeks	stabilize the progressive cognitive dysfunctions
Arcoverde, C. et al., 2014 [155]	treadmill	Moderate	2 times/week × 30 min × 16 weeks	improvement in the functional capacity
McCurry, S.M. et al., 2011 [157]	walking	unclear	3 times/week × 6 months	improving sleep
Yang, S.Y. et al., 2015 [158]	cycling	Moderate	3 times/week × 40 min × 3 months	improve cognitive function
Hoffmann, K. et al., 2015 [160]	strength, cycling	Moderate-to-high	3 times/week × 60 min × 16 weeks	reduced neuropsychiatric symptoms
Yu, F. et al., 2021 [161]	cycling	Moderate-to-high	3 times/week × 20–50 min × 6 months	reduce decline in global cognition
Ahn, N. et al., 2015 [163]	resistance exercise	unclear	3 times/week × 5 months	improved muscle strength and endurance, cardiovascular function, and gait speed
Ben, A.I. et al., 2021 [164]	cycling, cognitive games	Moderate	Acute Exercise	improve cognitive functions
Sampaio, A. et al., 2019 [165]	Multicomponent Training	unclear	2 times/week × 45–55 min × 6 months	improve physical and cognitive functions
Rolland, Y. et al., 2007 [166]	Collective exercise program	Low	2 times/week × 60 min × 12 months	significantly slower decline in ADL score
Sanders, L. et al., 2020 [167]	walking and lower limb strength training	Low and high	3 times/week × 60 min × 24 weeks	no beneficial effects of the exercise vs. control group on cognitive function.
Toots, A. et al., 2017 [168]	Functional Exercise program	High	5 times/2 week × 45 min × 4 months	no superior effects on global cognition or executive function

**Table 2:** Characteristics and risk of bias in exercise intervention studies for Alzheimer’s disease.

Author (s)	Study Design	Sample Size	Statistical Method	Consistency of Results	Risk of Bias
Friedman, R. et al., 1991 [153]	Randomized, non-blinded two-group experimental	30	MANOVA	High	Medium
Venturelli, M. et al., 2011 [154]	Randomized controlled trial	21	ANOVA	High	Medium
Arcoverde, C. et al., 2014 [155]	Randomized controlled trial	20	independent sample *t*-test	High	Medium
McCurry, S.M. et al., 2011 [157]	Randomized, controlled trial with blinded assessors	132	unclear	High	Unclear
Yang, S.Y. et al., 2015 [158]	Randomized controlled trial	50	paired samples *t*-test	High	Low
Hoffmann, K. et al., 2015 [160]	Randomized controlled trial	200	linear regression models using generalized estimating equations	High	Low
Yu, F. et al., 2021 [161]	Randomized controlled trial	96	2-sided *t*-test	High	Low
Ahn, N. et al., 2015 [163]	Randomized controlled trial	23	unclear	High	Unclear
Ben, A.I. et al., 2021 [164]	Randomized controlled trial	79	ANOVA	High	Low
Sampaio, A. et al., 2019 [165]	Non-randomized study	37	ANOVA	High	Medium
Rolland, Y. et al., 2007 [166]	Randomized controlled trial	134	Multiple logistic regression analyses	High	Low
Sanders, L. et al., 2020 [167]	Randomized controlled trial	91	ANCOVA	Low	Medium
Toots, A. et al., 2017 [168]	Randomized controlled trial	186	Linear mixed models	Low	Medium

## Data Availability

Not applicable.
